# A randomized controlled protocol on the effect of moxibustion on the cardiac function and quality of life in patients with chronic heart failure

**DOI:** 10.1097/MD.0000000000026860

**Published:** 2021-08-13

**Authors:** Jierong He, Lihong Jiao, Miao Xu, Rui Gong, Zhengyv Guo

**Affiliations:** a Deyang Vocational College of Technology and Trade, Deyang, Sichuan province, China; b Tianjin University of Traditional Chinese Medicine, Tianjin, China; c pixianNO.4 Middle School, Chengdu, Sichuan province, China.

**Keywords:** chronic heart failure, moxibustion, protocol, randomized controlled trial

## Abstract

**Background:**

Chronic heart failure (CHF) is the final result of various cardiovascular diseases, with high morbidity and high mortality, which seriously threaten people's health and quality of life. It has become a public health problem in the world. There is currently no specific treatment. Moxibustion, as a complementary and replacement therapy, has advantages in the treatment of chronic heart failure, but it lacks standard clinical studies to verify it. Therefore, the purpose of this randomized controlled trial is to evaluate the effect of moxibustion on the heart function and quality of life of patients with CHF.

**Methods:**

This is a prospective randomized controlled trial to study the effect of moxibustion on the heart function and quality of life of patients with CHF. This is approved by the clinical research ethics committee of our hospital. Patients were randomly divided into observation group (moxibustion combined with Western medicine treatment group) or control group (conventional Western medicine treatment group). There is a follow-up for 3 months after 6 weeks of treatment. Observation indicators include total effective rate of cardiac function improvement, Minnesota Living with Heart Failure Questionnaire , left ventricular ejection fraction , N-terminal pro-brain natriuretic peptide , 6-minute walk test , adverse reactions, etc. Data were analyzed using the statistical software package SPSS version 18.0 (Chicago, IL).

**Discussion:**

This study will evaluate the clinical efficacy of moxibustion in the treatment of CHF. The results of this study will provide a reliable reference for the clinical choice of moxibustion as an adjuvant treatment for chronic heart failure.

**Trial registration:**

OSF Registration number: DOI 10.17605/OSF.IO/29XE7.

## 1 Introduction

Chronic heart failure is the final result of various cardiovascular diseases. It has the characteristics of high morbidity, high mortality, and high rehospitalization rate. It affects at least 26 million people worldwide and seriously threatens the lives and health of patients. It has become a world public health problem.^[1,2]^ According to data from 2019, in the United States, an estimated 6.2 million adults suffer from chronic heart failure (CHF). Among adults 65 years of age and older, 21 out of 1000 elderly people are affected by heart failure.^[3]^ Although the current treatment strategy for CHF has made progress, there are a variety of treatment options, such as angiotensin-converting enzyme inhibitors , angiotensin receptor blockers , beta blockers and aldosterone antagonists, coronary revascularization, implantable cardioverter-defibrillators, etc, but the results are not satisfactory.^[4]^ The latest data from an international prospective cohort study of congestive heart failure show that the total mortality rate of CHF within 1 year is still 16.5%.^[5]^ Therefore, it is still necessary to explore complementary and alternative therapies to optimize the treatment strategy of CHF.

Moxibustion is an external TCM treatment based on the meridian theory. It has been widely used in the treatment of various diseases including cardiovascular diseases and has shown good curative effects.^[6,7]^ The curative effect of moxibustion mainly comes from the thermal effect of moxa burning on acupoints on the body surface,^[8]^ and the chemical stimulation of the medicinal ingredients in the wormwood.^[9,10]^ Animal experiments have found that moxibustion plays a key role in myocardial protection by regulating neuroendocrine immune response, inhibiting excessive autophagy, and improving myocardial hypertrophy and cardiac function.^[11,12]^ The mechanism may be related to the upregulating of mTOR, inhibiting myocardial autophagy, and enhancing anti-inflammatory responses.^[13]^ Previous systematic reviews have confirmed that moxibustion or acupuncture combined with conventional Western medicine is better than traditional treatment group,^[14]^ but due to factors such as limited research quality, small sample size, and short follow-up time, there is still no reliable clinical evidence to verify moxibustion as a complementary and alternative therapy on the heart function and quality of life of patients with chronic heart failure. Therefore, we plan to evaluate the effectiveness and safety of moxibustion on chronic heart failure through this randomized controlled trial.

## 2 Materials and methods

### 2.1 Study design

**Figure 1:**
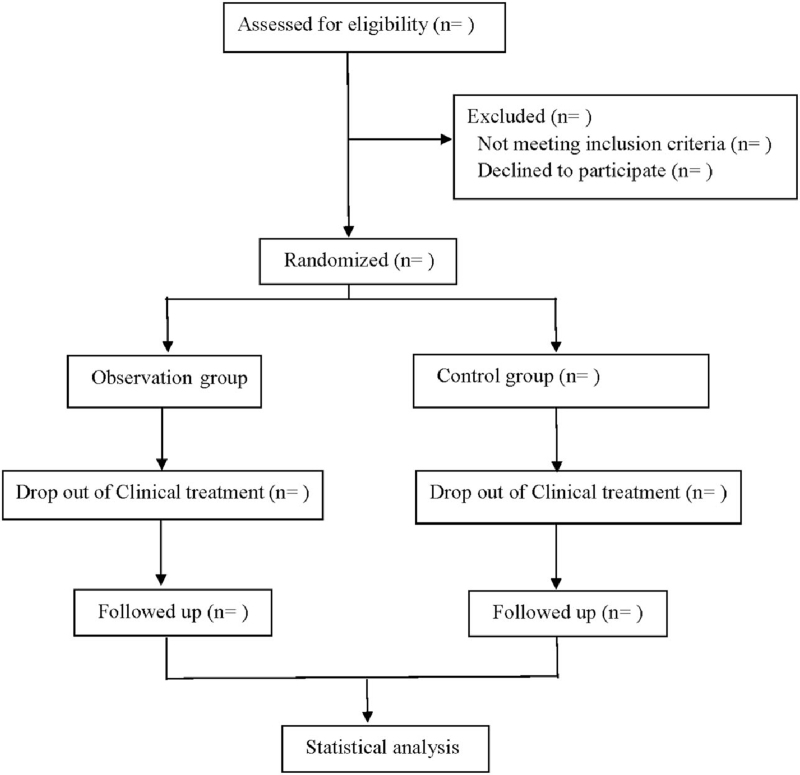
Flow diagram.

This is a prospective randomized controlled trial to study the clinical efficacy of moxibustion in the treatment of chronic heart failure. This study will follow the comprehensive trial reporting standards,^[15]^ and the flowchart is shown in Figure 1.

### 2.2 Ethics and registration

This research protocol complies with the Declaration of Helsinki and passed the review of the clinical research ethics committee of our hospital. This experiment has been registered in (registration number: DOI 10.17605/OSF.IO/29XE7). Before randomization, all patients were required to sign an informed consent form, and they can choose whether to continue the trial at any time.

### 2.3 Sample size

The calculation of sample size is based on the mean and standard deviation of Minnesota Living with Heart Failure Questionnaire ^[16]^ score after the end of treatment. According to the results of the pilot study, the observation group was 29.26 ± 5.26 and the control group was 32.82 ± 6.32. Set up α = 0.025, 1-sided test, β = 0.20. The PASS15.0 software calculates that each group requires 58 participants, and the estimated dropout rate is 10%. Each group will include 65 participants.

### 2.4 Patients

Inclusion criteria: Meet the diagnostic criteria of chronic heart failure,^[17]^ Doppler ultrasound showed left ventricular ejection fraction <50%^[18]^; The age ranged from 18 to 75 years old; New York Heart Association (NYHA) grades III to IV; Patients had strong compliance and signed an informed consent form.

Exclusion criteria: Patients with heart failure induced by other diseases such as congenital heart disease; Combined with other serious diseases such as malignant tumors, etc; Other heart diseases such as severe arrhythmia, etc; Combined with severe mental diseases; For this study, patients who are allergic to medication or have contraindications; Patients with skin ulceration at the moxibustion site.

### 2.5 Study design

Eligible participants were randomly assigned to the treatment group or the control group at a ratio of 1:1 using a random tool based on the central network. An independent statistician who was not involved in the implementation of the experiment or statistical analysis used SAS 9.3 software (SAS Institute, Cary, NC) to generate random sequences. The clinical research coordinator entered the participant's information on the tablet and was given a random number. The research assistant gets the participant's assignment from the computer. Throughout the research process, the research assistant is responsible for screening, recruiting participants, and assigning random numbers to the participants who have been included. Considering the operational nature of the intervention method, participants and operating physicians may be aware of random allocation. However, the evaluators of research results and the statisticians of data statistics and analysis have no knowledge of the distribution.

### 2.6 Intervention

#### 2.6.1 Moxibustion treatment

The patient takes the supine position and fully exposes the acupoints. The acupoints of moxibustion are: Qihai, Guanyuan, Bilateral Zusanli, Bilateral Bloodhai. Cut the moxa stick (1.8 × 20 cm, Nanyang Hanyi Moxa Co Ltd, Nanyang, China) into 5 cm pieces, put it in a moxibustion box (23 × 16 × 9.2 cm, Nanyang Hanyi Moxa Co) after lighting, and then the moxibustion box is placed on the selected acupuncture point. The moxa was ignited on the iron gauze, which is in the lower part of the box. And the lid is replaced. If the patient reported a sensation of burning, the moxibustion box was opened or the box briefly lifted just above the skin, then put back in place. Remove the moxibustion box after 30 minutes. The whole procedure was performed in accordance with the State Standard of the People's Republic of China Manipulation of Acupuncture and Moxibustion: Part 1 Moxibustion (GB/T 21709.1-2008).^[19]^ The treatment time was 30 minutes every other day.

#### 2.6.2 Conventional Western medicine treatment

Oral spironolactone tablets (Zhongxin Pharmaceutical, Guoyao Zhunzi H10910011, Beijing, China), 20 mg each time, once a day; oral benazepril hydrochloride tablets (Novartis Pharmaceutical, Guoyao Zhunzi H20000292, Beijing, China), 20 mg each time, once a day; oral bisoprolol fumarate tablets (Yuandong Pharmaceutical, Guoyao Zhunzi H20083008, Chengdu, China), 10 mg each time, once a day; Digoxin tablets (Hangzhou Minsheng Pharmaceutical, Guoyao Zhunzi H133021657, Hangzhou, China), 0.5 mg each time, once a day.

The observation group was treated with moxibustion combined with Western medicine; the control group was treated with Western medicine alone. Both groups of patients completed 6 weeks of standard treatment.

### 2.7 Evaluation criteria and judgment of curative effect

#### 2.7.1 Primary outcomes

Total effective rate of cardiac function improvement (refer to the principle of clinical research on treating heart failure with new Chinese Medicine),^[20]^ Excellent: heart failure was essentially ameliorated or the NYHA classification increased by at least 2 levels; Valid: NYHA classification increased by 1 level; Invalid: NYHA classification remained the same before and after the treatment; Worsened: NYHA classification decreased by at least. Minnesota Living with Heart Failure Questionnaire .^[16]^ The higher the score, the worse the patient's quality of life.

#### 2.7.2 Secondary outcomes

Left ventricular ejection fraction , N-terminal pro-brain natriuretic peptide , tumor necrosis factor-α , interleukin-6 , 6-minute walk test.

#### 2.7.3 Adverse reactions

Including liver and kidney function abnormalities and any uncomfortable symptoms (such as dizziness, nausea, etc) during treatment.

The above observation indicators were collected the day before and after treatment. All patients were followed up for 3 months, and data were collected according to the same standards in the first and third months.

### 2.8 Data collection and management

One or two assistants will collect and record the whole data. Personal information about potential participants and registered participants will be collected, shared, and kept in an independent storage room to protect confidentiality before, during, and after the test. The access to the database will be restricted to the researchers in this study team.

### 2.9 Statistical analysis

The collected data were statistically analyzed using SPSS 18.0 software. The enumeration data used the *χ*^2^test; the measurement data used the mean ± standard deviation ($\bar{x}$ ± S), the normal distribution used the independent sample *t* test, and the skewed distribution used the Mann-Whitney *U* test. When *P* < .05, the difference was considered statistically significant.

## 3 Discussion

Chronic heart failure is the main reason for hospitalization of elderly people worldwide, and has a negative impact on the quality of life of patients, with a high mortality rate.^[21]^ Although medicine and technology have made progress in the treatment of heart failure, the mortality, morbidity, and treatment costs of chronic heart failure are still high and rising year by year.^[22]^ Therefore, it is urgent to explore safe and effective complementary and alternative therapies.

Traditional medicine believes that chronic heart failure is a chronic debilitating disease dominated by heart-yang deficiency, accompanied by blood stasis, drinking water, and turbid phlegm. The treatment should be to warm the yang to clear the collateral, promote qi and diuresis, and promote blood circulation.^[23]^ Moxibustion is an external treatment method based on the acupuncture meridian theory, used to prevent and treat various chronic diseases. The thermal effect of the burning of moxa sticks stimulates the conduction of acupoints through qi, which has the effect of warming yang and promoting the pulse, promoting qi, and activating blood.^[24]^ This study verified the auxiliary efficacy of moxibustion by observing the comparison of the curative effect of moxibustion combined with conventional Western medicine and simple conventional Western medicine, and further explored the effect of moxibustion on the heart function and quality of life of patients with chronic heart failure.

Since this study is a single-center study, the regionalization of the included population may have a certain impact on the results. Therefore, more randomized controlled studies with more centers and large samples are needed to verify our conclusions.

## Author contributions

**Conceptualization:** Jierong He and Miao Xu

**Data curation:** Jierong He and Lihong Jiao

**Funding acquisition:** Zhengyv Guo

**Software:** Lihong Jiao and Rui Gong

**Supervision:** Miao Xu and Rui Gong

**Writing – original draft:** Jierong He and Lihong Jiao

**Writing – review & editing:** Jierong He and Zhengyv Guo
